# Corrections to the Bekenstein–Hawking Entropy of the HNUTKN Black Hole Due to Lorentz-Breaking Fermionic Einstein–Aether Theory

**DOI:** 10.3390/e26040326

**Published:** 2024-04-11

**Authors:** Xia Tan, Cong Wang, Shu-Zheng Yang

**Affiliations:** 1 College of Physics and Electronic Engineering, Qilu Normal University, Jinan 250300, China; 20152329@qlnu.edu.cn; 2 College of Physics and Astronomy, China West Normal University, Nanchong 637002, China; szyang@cwnu.edu.cn

**Keywords:** hot NUT–Kerr–Newman black hole, Lorentz breaking, black hole entropy

## Abstract

A hot NUT–Kerr–Newman black hole is a general stationary axisymmetric black hole. In this black hole spacetime, the dynamical equations of fermions at the horizon are modified by considering Lorentz breaking. The corrections to the Hawking temperature and Bekenstein–Hawking entropy at the horizon of the black hole are studied in depth. Based on the semiclassical theory correction, the Bekenstein–Hawking entropy of this black hole is quantum-corrected by considering the perturbation effect of the Planck constant *ℏ*. The latter part of this paper presents a detailed discussion of the obtained results and their physical implications.

## 1. Introduction

It is well known that de Sitter spacetime can be thought of as being hot, since an observer moving along a geodesic will detect an isotropic background of thermal radiation with a temperature T. NUT–Kerr–Newman–de Sitter spacetimes are asymptotically de Sitter instead of being asymptotically flat. This is a more general axisymmetric background. We call the spacetime concerned here the NUT (Newman–Unti–Tamburino)–Kerr–Newman spacetime, since the de Sitter spacetime has been interpreted as being hot [1]. For brevity, we refer to hot NUT (Newman–Unti–Tamburino)–Kerr–Newman as HNUTKN. The purpose of the present paper is to study the corrections and physical implications of entropy for the hot NUT–Kerr–Newman (HNUTKN) black hole. Reference [2] investigates the Hawking radiation of Dirac particles in the spacetime, obtaining a pure thermal radiation spectrum. However, the actual quantum tunneling radiation rate of the black hole is related to the Bekenstein–Hawking entropy change, and the thermal radiation spectrum of the black hole should not be a blackbody spectrum. Research on the correction of quantum tunneling radiation in HNUTKN black holes could lead to new expressions for the quantum tunneling rate, Hawking temperature, and Bekenstein–Hawking entropy of this black hole. Only by considering the real quantum tunneling radiation can we provide some explanation for the puzzle of the black hole information loss, thus making the research more physically meaningful. The research method for the entropy of HNUTKN black holes and the series of results obtained in this paper include corresponding results for the NUT–Kerr–Newman spacetime, NUT–Kerr–Newman–de Sitter spacetime, Kerr–Newman spacetime, and Kerr spacetime. Therefore, it is necessary to conduct in-depth research on the correction of entropy for HNUTKN black holes.

The first and second laws of black hole thermodynamics are both related to black hole entropy, which in turn is associated with black hole radiation, indicating a close connection to black hole information. Recent research results have shown that the thermal radiation spectrum of a black hole is no longer strictly a blackbody spectrum but contains information that escapes with the radiation, thereby implying that no information is lost during the process of black hole thermal radiation [3,4,5]. Unitarity is one of the fundamental principles of quantum theory, and information conservation is necessary to ensure the unitarity and probability conservation of quantum theory. As a black hole radiates, a portion of the information is carried away beyond the black hole, while another portion remains as remnants, called “ashes”, due to the black hole ceasing to emit particles towards the end of its radiation. The mass of these remnants, denoted as $M_{res}$, is equal to the minimum mass of the black hole $M_{min}$. The specific heat capacity of a black hole is related to its Hawking temperature and entropy. Recent research has indicated that the generalized uncertainty principle (GUP) can lead to the formation of black hole remnants [6]. Different black holes may possess distinct remnants. The existence of black hole remnants indicates that black holes do not evaporate completely, as predicted by classical theory. In fact, there are still many unresolved issues regarding the remnants and the information paradox.

The calculation methods of black hole entropy include the brick wall model and the membrane model. ’t Hooft proposed the brick wall model in 1985 to explain black hole entropy and suggested that the entropy of a quantum gas (Hawking radiation) in thermal equilibrium with the black hole outside is equal to the black hole’s entropy [7]. Following this calculation method, Zhao et al. improved ’t Hooft’s brick wall model to obtain the membrane model and used this model to study black hole entropy [8,9,10]. According to the viewpoint of the membrane model, black hole entropy is actually the entropy of two-dimensional membranes on the black hole event horizon, which are curved surfaces intersecting the three-dimensional space at the same time. This entropy is contributed by the two-dimensional quantum gas on the event horizon, requiring the inclusion of a thin layer outside the black hole event horizon with a thickness ε and a distance *d* from the event horizon when calculating black hole entropy. By studying the entropy of the quantum gas in this thin layer and taking the limits ε→0 and d→0, the black hole entropy can be obtained. Research results show that the Bekenstein–Hawking entropy of a black hole is proportional to its event horizon area *A*, i.e., $S_{BH}\propto A/4$. For a HNUTKN black hole, according to the first law of black hole thermodynamics, its general expression in differential form is $dM=TdS_{BH}+\Omega dJ+QdV$, where *M* represents the Bekenstein–Smarr mass. By studying the quantum tunneling radiation of a black hole, the Hawking temperature at the event horizon can be determined. Since the relationship between the Hawking temperature T and the surface gravity κ of the event horizon is given by T=κ/2π, the study of the quantum tunneling radiation characteristics and the first law of black hole thermodynamics can be employed to investigate the Bekenstein–Hawking entropy of black holes. However, recent research on quantum gravity theories suggests that Lorentz relations need appropriate modifications in high-energy regimes. Before a theory of dispersion relations in the high-energy regime is established, it is generally believed that the order of magnitude of the correction terms for Lorentz dispersion relations is at the Planck scale [11,12,13,14,15]. The appearance of such correction terms has prompted investigations into the effects of Lorentz-breaking theories in high-energy physics on curved spacetime backgrounds and the modifications of the quantum tunneling radiation characteristics under specific curved spacetime backgrounds. In 2009, Horava proposed the Horava–Lifshitz gravity theory, which breaks the Lorentz symmetry by not having temporal and spatial symmetries in high-energy situations. Another Lorentz-breaking gravity theory is the Einstein–aether theory. The Horava–Lifshitz gravity theory and the Einstein–aether theory are consistent in low-energy situations. The effects of Lorentz-breaking on steady-state axisymmetric spacetime backgrounds are reflected in the spacetime metric [16]. Considering Lorentz breaking, modifications to the dynamical equations of bosons and fermions and in-depth studies of the quantum tunneling radiation characteristics of different black holes in specific curved spacetime backgrounds are topics worth exploring [17,18,19]. Through such research, we can delve into the entropy of black holes and gain a profound understanding of the physical significance of black hole entropy and information.

Section 2 below pertains to the modified forms of the dynamical equations of fermions with Lorentz breaking. Section 3 of this paper focuses on the research methods and physical significance of the obtained results on HNUTKN black hole entropy. Section 4 of this paper is a discussion of the research results.

## 2. Lorentz Breaking and the Modified Dynamical Equation of the Spinor Field

When considering the modification of the action of the spinor field with Lorentz-breaking fermionic Einstein–aether terms in a curved spacetime, one needs to consider the aether-like field vector $u^{\mu }$, the Chiral correction term, and the Carroll–Field–Jackiw (CFJ) correction term. After Carroll et al. proposed the CFJ correction term in 1990, research on modifications to Lorentz breaking has been carried out [20,21,22], and this aspect of research still has practical significance today. It is necessary to study the influence of Lorentz breaking on black hole quantum tunneling radiation and black hole entropy in different curved spacetimes. Before modifying the black hole entropy, one should first consider the effect of Lorentz breaking on the action of the spinor field. Introducing a Lorentz-breaking correction term in a curved spacetime can express the action of the spinor field as [17,23,24]

(1) $$\begin{array}{cc} S_{F} & =\int d^{4}x\sqrt{-g}{\bar{\psi }}_{\alpha_{1}\ldots \alpha_{k}}\left\{i\gamma^{\mu }\left(\partial_{\mu }-\frac{ie}{\hbar }A_{\mu }+i\Omega_{\mu }\right)\left[1-\frac{a^{\prime }\hbar^{2}}{m^{2}}\gamma^{\alpha }\left(\partial_{\alpha }-\frac{ie}{\hbar }A_{\alpha }+i\Omega_{\alpha }\right)\gamma^{\beta }\left(\partial_{\beta }-\frac{ie}{\hbar }A_{\beta }+i\Omega_{\beta }\right)\right]\right.\\ \; & \left.+\frac{\lambda }{m}{\left[u^{\alpha }\left(\partial_{\alpha }-ieA_{\alpha }+i\Omega_{\alpha }\right)\right]}^{2}+\frac{b^{\prime }}{\hbar m}\gamma^{5}-\frac{m}{\hbar }\right\}\psi_{\alpha_{1}\ldots \alpha_{k}} \end{array}$$

Here, $a^{\prime }$, $b^{\prime }$, and λ are all small dimensionless real numbers. $b^{\prime }$ is the coefficient for Chiral correction terms; $a^{\prime }$ is the coefficient for CFJ correction terms. $\gamma^{\mu },\gamma^{\alpha }$, and $\gamma^{\beta }$ are gamma matrices in a curved spacetime; $u^{\alpha }$ and $u^{\beta }$ are aether-like field vectors in a curved spacetime; and $u^{\mu }$ must satisfy $u^{\mu }u_{\mu }=\;\mathrm{const}$. $\gamma^{5}$ is the gamma matrix corresponding to the Chiral correction term. Since $\gamma^{5}$ appears in $S_{F}$, it is required that $\gamma^{5}$ is also a Hermitian matrix. $\Omega_{\mu },\Omega_{\alpha }$, and $\Omega_{\beta }$ are the spin connections. $\Omega_{\mu }=\frac{i}{2}\Gamma_{\mu }^{\alpha \beta }\Pi_{\alpha \beta },\Pi_{\alpha \beta }=\frac{i}{4}\left[\gamma^{\alpha },\gamma^{\beta }\right]$, where $\Gamma_{\mu }^{\alpha \beta }$ is the connection of Riemann space and is related to the metric tensor of the curved spacetime. $A_{\mu }\left(A_{\alpha },A_{\beta }\right)$ is the electromagnetic potential generated by a black hole charge. $\psi_{\alpha_{1}\ldots \alpha_{k}}$ represents the wave function of fermions. ${\bar{\psi }}_{\alpha_{1}\ldots \alpha_{k}}$ is the Hermitian conjugate of $\psi_{\alpha_{1}\ldots \alpha_{k}}$. The relationship between the gamma matrices in Equation (1) and the metric tensor in a curved spacetime is as follows:

(2) $$\begin{array}{c} \gamma^{\mu }\gamma^{\nu }+\gamma^{\nu }\gamma^{\mu }=2g^{\mu \nu }I \end{array}$$

(3) $$\begin{array}{c} \gamma^{\mu }\gamma^{5}+\gamma^{5}\gamma^{\mu }=0 \end{array}$$

where I is the identity matrix. According to the variational principle, we can derive the spinor field equations in a curved spacetime from Equation (1). Substituting $S_{F}$ into the following equation

(4) $$\begin{array}{c} \delta S_{F}=0, \end{array}$$

we obtain

(5) $$\begin{array}{cc} \; & \delta \left\{{\bar{\psi }}_{\alpha_{1}\cdots \alpha_{k}}\left[i\gamma^{\mu }\left(\partial_{\mu }-\frac{ie}{\hbar }A_{\mu }+i\Omega_{\mu }\right)\left[1-\frac{a^{\prime }\hbar^{2}}{m^{2}}\gamma^{\alpha }\gamma^{\beta }\left(\partial_{\alpha }-\frac{ie}{\hbar }A_{\alpha }+i\Omega_{\alpha }\right)\left(\partial_{\beta }-\frac{ie}{\hbar }A_{\beta }+i\Omega_{\beta }\right)\right]\right.\right.\\ \;\; & \left.\left.+\frac{\hbar \lambda }{m}+\frac{b^{\prime }}{\hbar m}\gamma^{5}-\frac{m}{\hbar }\right]\right\}\psi_{\alpha_{1}\cdots \alpha_{k}}=0 \end{array}$$

The equation shows that

(6) $$\begin{array}{c} \delta \left[\left(\frac{b^{\prime }}{\hbar m}\gamma^{5}-\frac{m}{\hbar }\right)\psi_{\alpha_{1}\cdots \alpha_{k}}\right]=\left(\frac{b^{\prime }}{\hbar m}\gamma^{5}-\frac{m}{\hbar }\right)\partial_{\mu }\psi_{\alpha_{1}\cdots \alpha_{k}}\delta x^{\mu } \end{array}$$

(7) $$\begin{array}{c} \delta {\left[u^{\alpha }\left(\partial_{\alpha }-\frac{ie}{\hbar }A_{\alpha }+i\Omega_{\alpha }\right)\right]}^{2}\psi_{\alpha_{1}\cdots \alpha_{k}}=u^{\alpha }u^{\beta }\left(\partial_{\alpha }-\frac{ie}{\hbar }A_{\alpha }+i\Omega_{\alpha }\right)\left(\partial_{\beta }-\frac{ie}{\hbar }A_{\beta }+i\Omega_{\beta }\right)\partial_{\mu }\psi_{\alpha_{1}\ldots \alpha_{k}}\delta x^{\mu } \end{array}$$

Noting that $\delta {\bar{\psi }}_{\alpha_{1}\ldots \ldots \alpha_{k}}$$=\partial \mu {\bar{\psi }}_{\alpha_{1}\ldots \ldots \alpha_{k}}\delta x^{\mu }$ and $\delta \psi_{\alpha_{1}\ldots \ldots \alpha_{k}}=\partial \mu \psi_{\alpha_{1}\ldots \ldots \alpha_{k}}\delta x^{\mu }$, the modified spinor field equation can be obtained as follows from Equations (5)–(7):

(8) $$\begin{array}{cc} \; & i\gamma^{\mu }\left(\partial_{\mu }-\frac{i\varphi }{\hbar }A_{\mu }+\Omega_{\mu }\right)\left[1-\frac{a^{\prime }\hbar^{2}}{m^{2}}\gamma^{\alpha }\gamma^{\beta }\left(\partial_{\alpha }-\frac{ie}{\hbar }A_{\alpha }+i\Omega_{\alpha }\right)\left(\partial_{\beta }-\frac{ie}{\hbar }A_{\beta }+i\Omega_{\beta }\right)\right]\psi_{\alpha_{1}\cdots \alpha_{k}}\\ \; & =\left[\frac{m}{\hbar }-\frac{b^{\prime }}{\hbar m}\gamma^{5}-\frac{\hbar \lambda }{m}u^{\alpha }u^{\beta }\left(\partial_{\alpha }-ieA_{\alpha }+i\Omega_{\alpha }\right)\left(\partial_{\beta }-ieA_{\beta }+i\Omega_{\beta }\right)\right]\psi_{\alpha_{1}\cdots \alpha_{k}}. \end{array}$$

The equation for ${\bar{\psi }}_{\alpha_{1}\ldots \ldots \alpha_{k}}$ is the Hermitian conjugate of Equation (8). Equation (8) is a modified form of the fermion dynamical equation. The expression $\psi_{\alpha_{1}\ldots \ldots \alpha_{k}}$ in this equation represents the wave function of an arbitrary spin fermion. This equation applies to any spin fermion. For spin-1/2 fermions, the wave function $\psi_{\alpha_{1}\ldots \alpha_{k}}$ in Equation (8) can be expressed using the WKB theory:

(9) $$\begin{array}{c} \psi_{\frac{1}{2}}=\left(\begin{array}{c} A\\ B \end{array}\right)e^{\frac{i}{\hbar }s_{a}} \end{array}$$

Here, $S_{a}$ is the action for spin-1/2 fermions, which refers to Dirac particles in a curved spacetime. In this case, Equation (8) is known as the modified form of the Dirac equation. For spin-3/2 fermions, the wave function in Equation (8) is denoted as $\psi_{\alpha_{1}\ldots \ldots \alpha_{k}}\to \psi_{\nu }$, and the equation is then referred to as the modified form of the Rarita–Schwinger equation. In studying the solutions of Equation (8) in this scenario, the following conditions must be satisfied:

(10) $$\begin{array}{c} \gamma^{\nu }\psi_{\nu }=0. \end{array}$$

In general, for fermions of any spin, the wave function in Equation (8) should be substituted as $\psi_{\alpha_{1}\ldots \ldots \alpha_{k}}$, and the corresponding additional condition for Equation (8) is

(11) $$\begin{array}{c} \gamma^{\mu }\psi_{\mu \alpha_{2}\ldots \ldots \alpha_{k}}=D_{\mu }{\psi^{\mu }}_{\alpha_{2}\ldots \ldots \alpha_{k}}={\psi^{\mu }}_{\mu \alpha_{3}\ldots \ldots \alpha_{k}}=0. \end{array}$$

When k=0, Equation (8) reduces to the modified form of the Dirac equation, in which case there are no additional conditions. When k=1, Equation (11) reverts back to Equation (10), and, in this scenario, Equation (8) represents the modified form of the Rarita–Schwinger equation. When studying the dynamical characteristics of fermions with different spins in a specific black hole spacetime, the methods and processes for solving are not the same. When analyzing the dynamical equations at the black hole horizon using semiclassical theory, condition (11) does not need to be considered, and the calculation of the quantum tunneling rates will yield the same results. To illustrate the application of Equation (8) more clearly, let us consider spin-1/2 fermions, i.e., Dirac particles. Substituting Equation (9) into Equation (8), we obtain the equation that the particle action $S_{a}$ must satisfy:

(12) $$\begin{array}{cc} \; & \left\{-i\gamma^{\mu }\left(\partial_{\mu }S_{a}-eA_{\mu }\right)\left[1+\frac{a^{\prime }}{m^{2}}g^{\alpha \beta }\left(\partial_{\alpha }S_{a}-eA_{\alpha }\right)\left(\partial_{\beta }S_{a}-eA_{\beta }\right)\right]\right.\\ \; & -m+\frac{b^{\prime }}{m}\gamma_{0}^{5}\left.+\frac{\lambda }{m}u^{\alpha }u^{\beta }\left(\partial_{\alpha }S_{a}-eA_{\alpha }\right)\left(\partial_{\beta }S_{a}-eA_{\beta }\right)\right\}\left(\begin{array}{c} A\\ B \end{array}\right)=0 \end{array}$$

In this equation, several small terms, such as $\hbar \Omega_{\mu }$, $\hbar \Omega_{\alpha }$, and $\hbar \Omega_{\beta }$, have been neglected. Therefore, Equation (12) represents a semiclassical, Lorentz-breaking modified dynamical equation for spin-1/2 fermions, also known as the spin-1/2 spinor field equation. The matrices $g^{\alpha \beta }$ and $\gamma^{\alpha }\gamma^{\beta }$ in this matrix equation must satisfy Equation (2). $\gamma_{0}^{5}$ is closely related to $\gamma^{5}$, requiring $\gamma^{5}$ to satisfy Equation (3) and $\gamma_{0}^{5}$ to be a real number. Thus, it is required that $\gamma^{5}=\gamma_{0}^{5}I$, where I denotes the identity matrix. For the matrix Equation (12) to have solutions, the corresponding determinant of the matrices in the equation must be equal to zero, i.e.,

(13) $$\begin{array}{cc} \; & \gamma^{\mu }\left(\partial_{\mu }S_{a}-eA_{\mu }\right)\left[1+\frac{a^{\prime }}{m^{2}}g^{\alpha \beta }\left(\partial_{\alpha }S_{a}-eA_{\alpha }\right)\left(\partial_{\beta }S_{a}-eA_{\beta }\right)\right]\\ \; & =\frac{\lambda }{m}u^{\alpha }u^{\beta }\left(\partial_{\alpha }S_{a}-eA_{\alpha }\right)\left(\partial_{\beta }S_{a}-eA_{\beta }\right)+\frac{b^{\prime }}{m}\gamma_{0}^{5}-m=0 \end{array}$$

By multiplying both sides of this equation by $\gamma^{\nu }\left(\partial_{\nu }S-eA_{\nu }\right)$ and neglecting higher-order small terms, and using Equation (2), we can obtain

(14) $$\begin{array}{c} g^{\mu \nu }\left(1+2a^{\prime }\right)\left(\partial_{\mu }S_{a}-eA_{\mu }\right)\left(\partial_{\nu }S_{a}-eA_{\nu }\right)+2\lambda u^{\mu }u^{\nu }\left(\partial_{\mu }S_{a}-eA_{\mu }\right)\left(\partial_{\nu }S_{a}-eA_{\nu }\right)+2b^{\prime }\gamma_{0}^{5}-m^{2}=0 \end{array}$$

This equation describes the spinor field equation for spin-1/2 fermions, incorporating terms such as the CFJ correction term, aether-like correction term, and Chiral correction term. Equation (14) presents the modified dynamical equations for spin-1/2 fermions utilizing the particle action $S_{a}$, with three correction terms included. Equation (14) is applicable for general static, stationary, and non-stationary black hole spacetimes. Therefore, Equation (14) represents a semiclassical spinor field equation, which has been modified to incorporate Lorentz-breaking effects. It should be noted that for fermions of any spin, there are different matrices corresponding to the wave functions and different additional conditions. The modified form of the fermion dynamical equation is represented by the action $S_{a}$, as shown in Equation (14).

## 3. Research on the Entropy of HNUTKN Black Holes

The line element of the HNUTKN black hole spacetime in Boyer–Lindquist coordinates is expressed as

(15) $$\begin{array}{cc} dS^{2} & =\rho^{2}\left(\frac{dr^{2}}{\Delta_{r}}+\frac{1}{\Delta_{\theta }}d\theta^{2}\right)+\frac{\sin^{2}\theta }{\rho^{2}J^{2}}\Delta_{\theta }{\left[adt-\left(r^{2}+a^{2}\right)d\phi \right]}^{2}-\frac{\Delta_{r}}{\rho^{2}J^{2}}{\left[dt-\left(a-\frac{{\left(n-a\cos\theta \right)}^{2}}{a}\right)d\phi \right]}^{2} \end{array}$$

Here,

(16) $$\begin{array}{cc} \rho^{2} & =r^{2}+{\left(n-a\cos\theta \right)}^{2}=r^{2}+n^{2}-2an\cos\theta +a^{2}\cos^{2}\theta \\ \Delta_{r} & =\left(r^{2}+a^{2}-n^{2}\right)\left(1-\frac{1}{3}\Lambda r^{2}\right)-2Mr+Q^{2}\\ \Delta_{\theta } & =1+\frac{1}{3}\Lambda {\left(n-a\cos\theta \right)}^{2}\\ J & =1+\frac{1}{3}\Lambda \left(a^{2}-n^{2}\right) \end{array}$$

Here, Λ is the cosmological constant, *M* is the black hole mass, *Q* is the black hole charge, the NUT parameter is *n*, also known as the gravitational magnetic type mass, and the angular momentum parameter is *a*. Due to the electromagnetic charge *Q* of the black hole, the electromagnetic potentials $A_{\mu }$, $A_{t}$, and $A_{\phi }$ are expressed as

(17) $$\begin{array}{c} A_{\mu }=\left(-\frac{Qr}{\rho^{2}J},0,0,\frac{Qra\sin^{2}\theta }{\rho^{2}J}\right) \end{array}$$

According to Equations (15) and (16), the determinant corresponding to the metric $g_{\mu \nu }$ is obtained as

(18) $$\begin{array}{c} g=\left|g_{\mu \nu }\right|=-\frac{\sin^{2}\theta }{J^{4}}\left\{r^{2}+a^{2}-{\left[a^{2}-{\left(n-a\cos\theta \right)}^{2}\right]}^{2}\right\} \end{array}$$

Using Equation (18) and the algebraic cofactor ${\left(-1\right)}^{\mu +\nu }\Delta^{\mu \nu }$ of $g_{\mu \nu }$, the non-zero components of the contravariant metric tensor corresponding to the line element (15) can be, respectively, calculated as

(19) $$\begin{array}{cc} g^{tt} & =-\frac{J^{2}}{\rho^{2}}\left\{\frac{{\left(r^{2}+a^{2}\right)}^{2}}{\Delta_{r}}-\frac{1}{a^{2}\Delta_{\theta }\sin^{2}\theta }{\left[a^{2}-{\left(n-a\cos\theta \right)}^{2}\right]}^{2}\right\}\\ g^{rr} & =\frac{\Delta_{r}}{\rho^{2}}\\ g^{\theta \theta } & =\frac{\Delta_{\theta }}{\rho^{2}}\\ g^{\phi \phi } & =\frac{J^{2}}{\rho^{2}}\left(\frac{1}{\Delta_{\theta }\sin^{2}\theta }-\frac{a^{2}}{\Delta_{r}}\right)\\ g^{t\phi } & =\frac{J^{2}}{\rho^{2}}\left[\frac{a}{\Delta_{r}}\left(r^{2}+a^{2}\right)-\frac{1}{\Delta_{\theta }a\sin^{2}\theta }\left(a^{2}-{\left(n-a\cos\theta \right)}^{2}\right)\right] \end{array}$$

The Bekenstein–Hawking entropy of the HNUTKN black hole is related to the Hawking temperature at the black hole horizon. Therefore, it is necessary to determine the location of the black hole horizon. According to the null supersurface equation at the black hole horizon, it can be determined that

(20) $$\begin{array}{c} g^{\mu \nu }\frac{\partial F}{\partial x^{\mu }}\frac{\partial F}{\partial x^{\nu }}=0 \end{array}$$

The normal vector of the hypersurface $F\left(x^{\mu }\right)=0$ is $n_{\mu }$, where $n_{\mu }=\frac{\partial F}{\partial x^{\mu }}$. The definition of the null hypersurface is

(21) $$\begin{array}{c} n^{\mu }n_{\mu }=0 \end{array}$$

We have $g^{\mu \nu }n_{\mu }n_{\mu }=0$, so we have the null supersurface Equation (20). Substituting Equation (19) into Equation (20) and taking into account the stationary axisymmetric nature of the HNUTKN black hole, we obtain the equation satisfied by the event horizon $r_{H}$ or the cosmological horizon $r_{c}$ of this black hole as

(22) $$\begin{array}{c} g^{rr}=\frac{\Delta_{r}}{\rho^{2}}=0\to \Delta_{r}\to 0 \end{array}$$

Specifically,

(23) $$\begin{array}{c} \left(r^{2}+a^{2}-n^{2}\right)\left(1-\frac{1}{3}\Lambda r^{2}\right)-2Mr+Q^{2}=0 \end{array}$$

In order to solve Equation (14), it is necessary to correctly choose the aether-like vector field $u^{\mu }$ according to the line element (15) as follows:

(24) $$\begin{array}{cc} u^{t} & =\frac{c_{t}}{\sqrt{g_{tt}}},u^{t}u_{t}=u^{t}u^{t}g_{tt}=c_{t}^{2}\\ u^{r} & =\frac{c_{r}}{\sqrt{g_{rr}}},u^{r}u_{r}=u^{r}u^{r}g_{rr}=c_{r}^{2}\\ u^{\theta } & =\frac{c_{\theta }}{\sqrt{g_{\theta \theta }}},u^{\theta }u_{\theta }=u^{\theta }u^{\theta }g_{\theta \theta }=c_{\theta }^{2}\\ u^{\phi } & =\frac{c_{\phi }}{\sqrt{g_{\phi \phi }}},u^{\phi }u_{\phi }=u^{\phi }u^{\phi }g_{\phi \phi }=c_{\phi }^{2} \end{array}$$

Clearly, $u^{\mu }$ satisfies $u^{\mu }u_{\mu }=\;\mathrm{const}$. In order to determine $\gamma_{0}^{5}$ in Equation (14), we must choose $\gamma^{\mu }$ satisfying Equation (2) and the Hermitian matrix $\gamma^{5}$ satisfying Equation (3). Therefore, we choose the frame field $e_{I}^{\mu }$ and determine the gamma matrix $\gamma^{\mu }$ as follows:

(25) $$\begin{array}{c} \gamma^{\mu }=e_{I}^{\mu }\gamma^{I} \end{array}$$

As a result, we obtain

(26) $$\begin{array}{c} \gamma^{\mu }\gamma^{\nu }+\gamma^{\nu }\gamma^{\mu }=e_{I}^{\mu }\gamma^{I}e_{J}^{\nu }\gamma^{J}+e_{J}^{\nu }\gamma^{J}e_{I}^{\mu }\gamma^{I}=2g^{\mu \nu }I \end{array}$$

where $e_{I}^{\mu }e_{J}^{\nu }\eta^{IJ}=g^{\mu \nu }$, $e_{J}^{\nu }e_{I}^{\mu }\eta^{JI}=g^{\nu \mu }=g^{\mu \nu }$. Clearly, $\gamma^{\mu }$ satisfies condition (2). Here, the values of I and *J* are (0,1,2,3), corresponding to the coordinates (t,r,θ,ϕ). We choose $\gamma^{5}$ as follows:

(27) $$\begin{array}{cc} \gamma^{5} & =\frac{i\gamma_{0}^{5}}{4!}\varepsilon_{\mu \nu k\lambda }\gamma^{\mu }\gamma^{\nu }\gamma^{k}\gamma^{\lambda }\\ \; & =\frac{i\gamma_{0}^{5}}{4!}\varepsilon_{IJKL}e_{\mu }^{I}e_{\nu }^{J}e_{k}^{I}e_{\lambda }^{J}e_{I}^{\mu },\gamma^{I^{\prime }}e_{I}^{\nu }\gamma^{J^{\prime }}e_{k^{\prime }}^{k}\gamma^{k^{\prime }}e_{L}^{\lambda },\gamma^{L^{\prime }}\\ \; & =\frac{i\gamma_{0}^{5}}{4!}\varepsilon_{IJKL}\gamma^{I}\gamma^{5}\gamma^{k}\gamma^{L}\\ \; & =i\gamma_{0}^{5}\gamma^{0}\gamma^{1}\gamma^{2}\gamma^{3}\\ \; & =\gamma_{0}^{5}\left(\begin{array}{cc} 0 & I\\ I & 0 \end{array}\right) \end{array}$$

Here, $\gamma_{0}^{5}$ is a real number. Clearly, $\gamma^{5}$ is a Hermitian matrix. $\varepsilon_{IJKL}$ is the Levi–Civita symbol, and $\varepsilon_{0123}=1$. Therefore, from Equations (25) and (27), we can obtain

(28) $$\begin{array}{c} e_{I}^{\mu }\gamma^{I}\gamma^{5}+\gamma^{5}e_{I}^{\mu }\gamma^{I}=0 \end{array}$$

(29) $$\begin{array}{c} \gamma^{\mu }\gamma^{5}+\gamma^{5}\gamma^{\mu }=e_{I}^{\mu }\gamma^{I}\gamma^{5}+\gamma^{5}e_{I}^{\mu }\gamma^{I}=e_{I}^{\mu }\left(\gamma^{I}\gamma^{5}+\gamma^{5}\gamma^{I}\right)=0 \end{array}$$

From these two equations, it can be seen that choosing $\gamma^{5}$ satisfies condition (3). The correct selection of $\gamma^{5}$, $\gamma^{\mu }$, and $u^{\mu }$ ensures the scientific validity of Equation (14). Substituting Equations (17), (18), (24), and (27) into Equation (14), we obtain the dynamic equation for spin-1/2 fermions in the HNUTKN black hole spacetime as follows:

(30) $$\begin{array}{cc} \; & \left(1+2a^{\prime }+2\lambda c_{t}^{2}\right)J^{2}\left\{\left[\frac{{\left(a^{2}-{\left(n-a\cos\theta \right)}^{2}\right)}^{2}}{a^{2}\Delta_{\theta }\sin^{2}\theta }-\frac{{\left(r^{2}+a^{2}\right)}^{2}}{\Delta_{r}}\right]\left[{\left(\frac{\partial S_{a}}{\partial t}\right)}^{2}\rho^{2}+\frac{2eQr}{J}\frac{\partial S_{a}}{\partial_{t}}+\frac{e^{2}Q^{2}r^{2}}{\rho^{2}J^{2}}\right]\right.\\ \; & \left.+2\left[\frac{a\left(r^{2}+a^{2}\right)}{\Delta_{r}}-\frac{a^{2}-{\left(n-a\cos\theta \right)}^{2}}{\Delta_{\theta }a\sin^{2}\theta }\right]\left[\left(\frac{\partial S_{a}}{\partial t}\right)\left(\frac{\partial S_{a}}{\partial_{\phi }}\right)\rho^{2}-\frac{eQra\sin^{2}\theta }{J}\frac{\partial S_{a}}{\partial_{t}}+\frac{eQr}{J}\frac{\partial S_{a}}{\partial_{\phi }}-\frac{e^{2}Q^{2}r^{2}a\sin^{2}\theta }{\rho^{2}J^{2}}\right]\right.\\ \; & \left.+\left(\frac{1}{\Delta_{\theta }\sin^{2}\theta }-\frac{a^{2}}{\Delta_{r}}\right)\left[{\left(\frac{\partial S_{a}}{\partial \phi }\right)}^{2}\rho^{2}-\frac{2\epsilon Qra\sin^{2}\theta }{J}\frac{\partial S_{a}}{\partial_{\phi }}+\frac{e^{2}Q^{2}r^{2}a^{2}\sin^{4}\theta }{\rho^{2}J^{2}}\right]\right\}+4\lambda \rho^{4}Y_{0}\\ \; & +\left(1+2a^{\prime }+2\lambda c_{r}^{2}\right)A_{r}\rho^{2}{\left(\frac{\partial S_{a}}{\partial_{r}}\right)}^{2}+\left(1+2a^{\prime }+2\lambda c_{\theta }^{2}\right)\rho^{2}{\left(\frac{\partial S_{a}}{\partial_{\theta }}\right)}^{2}=0 \end{array}$$

where

(31) $$\begin{array}{cc} Y_{0}= & u^{t}\left(\frac{\partial S_{a}}{\partial t}-eA_{t}\right)\left(u^{r}\frac{\partial S_{a}}{\partial r}+u^{\theta }\frac{\partial S_{a}}{\partial \theta }\right)+u^{r}\frac{\partial S_{a}}{\partial r}\left[u^{\theta }\frac{\partial S_{a}}{\partial \theta }+u^{\phi }\left(\frac{\partial S_{a}}{\partial \phi }-eA_{\phi }\right)\right]\\ \; & +u^{\theta }u^{\phi }\left(\frac{\partial S_{a}}{\partial \theta }\right)\left(\frac{\partial S_{a}}{\partial \phi }-eA_{\phi }\right)+2b^{\prime }\gamma^{5}-m^{2} \end{array}$$

In Equation (30), the sum of the four terms related to $\frac{1}{\Delta_{\theta }\sin^{2}\theta \rho^{2}}$ is zero. Multiplying both sides of this equation by $\frac{1}{\rho^{2}}$ yields

(32) $$\begin{array}{cc} \; & \left(1+2a+2\lambda c_{t}^{2}\right)J^{2}\left\{\left[\frac{{\left(a^{2}-{\left(n-a\cos\theta \right)}^{2}\right)}^{2}}{a^{2}\Delta_{\theta }\sin^{2}\theta }-\frac{{\left(r^{2}+a^{2}\right)}^{2}}{\Delta_{r}}\right]\left[{\left(\frac{\partial S_{a}}{\partial t}\right)}^{2}+\frac{2eQr}{\rho^{2}J}\frac{\partial S_{a}}{\partial t}\right]\right.\\ \; & +2\left[\frac{a\left(r^{2}+a^{2}\right)}{\Delta_{r}}+\frac{a^{2}-{\left(n-a\cos\theta \right)}^{2}}{\Delta_{\theta }a\sin^{2}\theta }\right]\left[\left(\frac{\partial S_{a}}{\partial t}\right)\left(\frac{\partial S_{a}}{\partial_{\phi }}\right)-\frac{eQra\sin^{2}\theta }{\rho^{2}J}\frac{\partial S_{a}}{\partial t}+\frac{eQr}{\rho^{2}J}\frac{\partial S_{a}}{\partial \phi }\right]\\ \; & +\left(\frac{1}{\Delta_{\theta }\sin^{2}\theta }-\right.\left.\left.\frac{a^{2}}{\Delta_{r}}\right)\left[{\left(\frac{\partial S_{a}}{\partial t}\right)}^{2}-\frac{2eQra\sin^{2}\theta }{\rho^{2}J}\frac{\partial S_{a}}{\partial \phi }\right]-\frac{e^{2}Q^{2}r^{2}{\left(r^{2}+a^{2}+a^{2}\sin^{2}\theta \right)}^{2}}{\Delta_{r}\rho^{4}J^{2}}\right\}\\ \; & \left.+(1+2a+2\lambda c_{r}^{2}\right)A_{r}{\left(\frac{\partial S_{a}}{\partial r}\right)}^{2}+\left(1+2a+2\lambda c_{\theta }^{2}\right){\left(\frac{\partial S_{a}}{\partial \theta }\right)}^{2}+4\lambda \rho^{2}Y_{0}=0 \end{array}$$

Separate the variables in the equation, letting

(33) $$\begin{array}{c} S_{a}=-wt+R\left(r\right)+\Theta \left(\theta \right)+J_{0}\phi \end{array}$$

where *w* is the energy of the particle and $J_{0}$ is the component of the particle’s generalized momentum in the ϕ direction, with $J_{0}$ being a constant. The ability to separate $S_{a}$ into the form of Equation (33) is mainly due to the existence of two Killing vectors in the HNUTKN spacetime, namely ${\left(\frac{\partial }{\partial t}\right)}^{\alpha }$ and ${\left(\frac{\partial }{\partial \phi }\right)}^{\alpha }$. Substituting Equation (33) into Equation (32) and multiplying both sides of this equation by $\Delta_{r}$, noting that ${\left.\Delta_{r}\right|}_{r\to r_{H}}=0$, we obtain the form of the radial motion equation for the particle at the event horizon of this black hole as

(34) $$\begin{array}{cc} {\left[\Delta_{r}^{2}{\left(\frac{dR}{dr}\right)}^{2}\right]}_{r\to r_{H}} & =\frac{J^{2}\left(1+2a^{\prime }+2\lambda c_{t}^{2}\right)}{1+2a^{\prime }+2\lambda c_{r}^{2}}\left[{\left(r_{H}^{2}+a^{2}\right)}^{2}{\left(\frac{\partial S_{a}}{\partial t}\right)}^{2}+2a\left(r^{2}+a^{2}\right)\frac{\partial S_{a}}{\partial t}\frac{\partial S_{a}}{\partial \phi }+a^{2}{\left(\frac{\partial S_{a}}{\partial \phi }\right)}^{2}\right.\\ \; & \left.+\frac{2eQr{\left(r^{2}+a^{2}\right)}^{2}}{J\left(r^{2}+a^{2}+n^{2}\right)}\frac{\partial S_{a}}{\partial t}+\frac{2aeQr\left(r^{2}+a^{2}\right)}{J\left(r^{2}+a^{2}+n^{2}\right)}\frac{\partial S_{a}}{\partial \phi }+\frac{e^{2}Q^{2}r^{2}{\left(r^{2}+a^{2}\right)}^{2}}{J^{2}{\left(r^{2}+a^{2}+n^{2}\right)}^{2}}\right]\\ \; & =\frac{J^{2}\left(1+2a^{\prime }+2\lambda c_{t}^{2}\right)}{1+2a^{\prime }+2\lambda c_{r}^{2}}{\left[\left(r_{H}^{2}+a^{2}\right)\frac{\partial S_{a}}{\partial t}+a\frac{\partial S_{a}}{\partial \phi }+\frac{eQr\left({r_{H}}^{2}+a^{2}\right)}{J\left({r_{H}}^{2}+a^{2}+n^{2}\right)}\right]}^{2}\\ \; & =\frac{J^{2}\left(1+2a^{\prime }+2\lambda c_{t}^{2}\right)}{1+2a^{\prime }+2\lambda c_{r}^{2}}{\left({r_{\nu }}^{2}+a^{2}\right)}^{2}{\left(\omega -\omega_{0}\right)}^{2} \end{array}$$

where

(35) $$\begin{array}{cc} \omega_{0} & =\frac{aJ_{0}}{r_{H}^{2}+a^{2}}+\frac{eQr_{H}}{J{\left(r_{H}^{2}+a^{2}+n^{2}\right)}^{2}}\\ \; & =\frac{aJ_{0}J\left(r_{H}^{2}+a^{2}+n^{2}\right)+eQr_{H}\left(r_{H}^{2}+a^{2}\right)}{J\left(r_{H}^{2}+a^{2}\right)\left(r_{H}^{2}+a^{2}+n^{2}\right)} \end{array}$$

Based on Equation (34), it can be inferred that the radial component R(r) of the particle’s generalized momentum satisfies the following equation at the event horizon of this black hole:

(36) $$\begin{array}{c} {\left.\frac{dR^{\pm }}{dr}\right|}_{r\to r_{H}}=\pm \frac{\left(r_{H}^{2}+a^{2}\right)J}{{\left.\Delta_{r}\right|}_{r\to r_{H}}}{\left(\frac{1+2a^{\prime }+2\lambda c_{t}^{2}}{1+2a^{\prime }+2\lambda c_{r}^{2}}\right)}^{\frac{1}{2}}\left(\omega -\omega_{0}\right). \end{array}$$

According to the residue theorem, from Equation (36), we obtain the radial component of the particle’s generalized momentum as

(37) $$\begin{array}{c} R^{\pm }{|}_{r\to r_{H}}=\pm i\pi \frac{J\left(r_{H}^{2}+a^{2}\right)}{2\left[r_{H}-m+\frac{1}{3}\Lambda r_{H}\left(a^{2}-n^{2}\right)\right]}{\left(\frac{1+2a^{\prime }+2\lambda c_{t}^{2}}{1+2a^{\prime }+2\lambda c_{r}^{2}}\right)}^{\frac{1}{2}}\left(\omega -\omega_{0}\right) \end{array}$$

Let $R^{\pm }{|}_{r\to r_{H}}=R^{\pm }$; based on the WKB approximation theory and the black hole quantum tunneling radiation theory, the expression for the quantum tunneling rate of spin-1/2 fermions at the event horizon $r_{H}$ of the black hole is

(38) Γ∼exp−2ImSSa±=exp−2ImR±=exp−2πJrH2+a2rH−m+13ΛrHa2−n21+2a′+2λct21+2a′+2λcr212ω−ω0=exp−ω−ω0TH

where,

(39) $$\begin{array}{c} T_{H}=\frac{r_{H}-m+\frac{1}{3}\Lambda r_{H}\left(a^{2}-n^{2}\right)}{2\pi J\left(r_{H}^{2}+a^{2}\right)}{\left(\frac{1+2a^{\prime }+2\lambda c_{r}^{2}}{1+2a^{\prime }+2\lambda c_{t}^{2}}\right)}^{\frac{1}{2}} \end{array}$$

Here, $T_{H}$ is the Hawking temperature at the event horizon of the HNUTKN black hole, and the expression for this Hawking temperature contains Lorentz-breaking correction terms. The coefficient for the CFJ correction term is $a^{\prime }$, and the coefficient for the aether-like correction term is λ. The coefficient $b^{\prime }$ for the Chiral correction term does not appear in the expressions for Γ and $T_{H}$. This is due to $\Delta_{r}\left(r_{H}\right)\left(2b^{\prime }\gamma_{0}^{5}-m^{2}\right)=0$ in Equation (32), so $b^{\prime }$ and *m* do not appear in the expressions for Γ and $T_{H}$. For fermions with any other spin, the coefficient $b^{\prime }$ for the Chiral correction term also does not appear in the expressions for Γ and $T_{H}$. Equations (37)–(39) are all results obtained in the semiclassical theory. We can use $\Delta S_{BH}$ to represent the Bekenstein–Hawking entropy of this black hole and represent the change in Bekenstein–Hawking entropy of this black hole as Δ$S_{BH}$, so the expression for the quantum tunneling rate of this black hole (38) can be rewritten as

(40) $$\begin{array}{c} \Gamma =e^{\left(\Delta S_{BH}\right)} \end{array}$$

The quantity $S_{BH}$ is closely related to the first law of thermodynamics for black holes. The mathematical relationship between $S_{BH}$ and the black hole mass *M*, black hole angular velocity Ω, black hole charge *Q*, and black hole Hawking temperature $T_{H}$ can be expressed as

(41) $$\begin{array}{cc} dS_{BH} & =\frac{dM-\Omega dJ-QdV}{T_{H}}\\ \; & =\frac{dM-\Omega dJ-QdV}{T_{h}}{\left(\frac{1+2a^{\prime }+2\lambda c_{t}^{2}}{1+2a^{\prime }+2\lambda c_{r}^{2}}\right)}^{\frac{1}{2}} \end{array}$$

where $T_{h}$ is the Hawking temperature of this black hole without Lorentz-breaking correction terms. The expression for $T_{h}$, as given by Equation (39), is as follows:

(42) $$\begin{array}{c} T_{h}=\frac{r_{H}-m+\frac{1}{3}\Lambda r_{H}\left(a^{2}-n^{2}\right)}{2\pi J\left(r_{H}^{2}+a^{2}\right)} \end{array}$$

Therefore, if we denote the Bekenstein–Hawking entropy as $S_{bh}$, the integral form of Equation (41) can be rewritten as

(43) $$\begin{array}{c} S_{BH}={\left(\frac{1+2a^{\prime }+2\lambda c_{t}^{2}}{1+2a^{\prime }+2\lambda c_{r}^{2}}\right)}^{\frac{1}{2}}\int dS_{bh} \end{array}$$

According to Equation (15), it can be inferred that the two-dimensional line element on the event horizon of this black hole is given by

(44) $$\begin{array}{cc} d\sigma^{2} & =\frac{\rho^{2}}{\Delta_{\theta }}d\theta^{2}+\left[\frac{\Delta_{\theta }\sin^{2}\theta }{\rho^{2}J^{2}}{\left(r_{H}^{2}+a^{2}\right)}^{2}-\frac{\Delta_{r}\left(r_{H}\right)}{J^{2}\rho^{2}}{\left(a-\frac{{\left(n-a\cos\theta \right)}^{2}}{a}\right)}^{2}\right]d\phi^{2}\\ \; & =\frac{\rho^{2}}{\Delta_{\theta }}d\theta^{2}+\frac{\Delta_{\theta }\sin^{2}\theta }{\rho^{2}J^{2}}{\left(r_{H}^{2}+a^{2}\right)}^{2}d\phi^{2} \end{array}$$

Therefore, it can be seen that the determinant $g_{\theta \phi }$ corresponding to the two-dimensional covariant metric here is

(45) $$\begin{array}{c} g_{\theta \phi }=\frac{\sin^{2}\theta }{J^{2}}{\left(r_{H}^{2}+a^{2}\right)}^{2} \end{array}$$

Thus, the area of the event horizon of this black hole is given by

(46) $$\begin{array}{c} A_{EH}=\int \sqrt{g_{\theta \phi }}d\theta d\phi =\int \begin{array}{c} 2\pi \\ 0 \end{array}\int_{0}^{\pi }\frac{\sin^{2}\theta }{J^{2}}{\left(r_{H}^{2}+a^{2}\right)}^{2}d\theta d\phi =\frac{4\pi }{J}\left(r_{H}^{2}+a^{2}\right) \end{array}$$

The Bekenstein–Hawking entropy of this black hole is proportional to the area of its event horizon and can be expressed as

(47) $$\begin{array}{c} S_{bh}=\frac{\pi }{J}\left(r_{H}^{2}+a^{2}\right) \end{array}$$

Therefore, Equation (43) can be rewritten as

(48) $$\begin{array}{c} S_{BH}=\frac{\pi }{J}\left(r_{H}^{2}+a^{2}\right){\left(\frac{1+2a^{\prime }+2\lambda c_{t}^{2}}{1+2a^{\prime }+2\lambda c_{r}^{2}}\right)}^{\frac{1}{2}} \end{array}$$

This is the Bekenstein–Hawking entropy of the HNUTKN black hole in the framework of radius-metric theory, after Lorentz-breaking theory modifications. This is still a relevant result within the framework of radius-metric theory. Using quantum perturbation theory, we can make quantum corrections to the Bekenstein–Hawking entropy of this black hole [25,26,27,28]. In the framework of *ℏ* perturbation theory, the energy *E* and radial action R(r) of the quantum tunneling radiation particles can be expressed as

(49) $$\begin{array}{c} E=E_{0}+\hbar E_{1}+\hbar^{2}E_{2}+\cdots \end{array}$$

(50) $$\begin{array}{c} \tilde{R}\left(r\right)=R_{0}\left(r\right)+\hbar R_{1}\left(r\right)+\hbar^{2}R_{2}+\cdots \end{array}$$

$E_{0}$$=\left(\omega -\omega_{0}\right)$ corresponds to the energy in Equations (37) and (38). $R_{0}\left(r\right)$ satisfies Equations (34) and (36), and the expression for $R_{0}^{\pm }$ is given in Equation (37), which can be considered as $R_{0}\to R_{0}^{\pm }$, where $R_{0}^{\pm }=\pm \frac{i\pi J\left(r_{H}^{2}+a^{2}\right)}{2r_{H}-m+\frac{1}{3}\Lambda r_{H}\left(a^{2}-n^{2}\right)}{\left(\frac{1+2a^{\prime }+2\lambda c_{t}^{2}}{1+2a^{\prime }+2\lambda c_{r}^{2}}\right)}^{\frac{1}{2}}\left(\omega -\omega_{0}\right)$. Therefore, by expanding Equations (49) and (50) for $\hbar^{0}$, $\hbar^{1}$, $\hbar^{2}$, ⋯ and corresponding to different powers of *ℏ*, we can obtain

(51) $$\begin{array}{cc} {\left.\hbar^{0}:{\left(\frac{dR_{0}}{dr}\right)}^{2}\right|}_{r\to r_{H}} & =\frac{J^{2}{\left(r_{H}^{2}+a^{2}\right)}^{2}}{{\left.\Delta_{r}^{2}\right|}_{r\to r_{H}}}\left(\frac{1+2a^{\prime }+2\lambda c_{t}^{2}}{1+2a^{\prime }+2\lambda c_{r}^{2}}\right)E_{0}^{2},\\ {\left.\hbar^{1}:{\left(\frac{dR_{1}}{dr}\right)}^{2}\right|}_{r\to r_{H}} & =\frac{J^{2}{\left(r_{H}^{2}+a^{2}\right)}^{2}}{{\left.\Delta_{r}^{2}\right|}_{r\to r_{H}}}\left(\frac{1+2a^{\prime }+2\lambda c_{t}^{2}}{1+2a^{\prime }+2\lambda c_{r}^{2}}\right)E_{1}^{2}. \end{array}$$

From the equations satisfied by the radial actions corresponding to $\hbar^{n}$, it can be inferred that there exists a certain proportional relationship between $R_{i}^{\pm }$ and $R_{0}^{\pm }$. Therefore, the total energy *E* can be rewritten as

(52) $$\begin{array}{c} E=E_{0}+\sum_{i=1}^{\infty }\frac{\alpha_{i}\hbar^{i}}{S_{bh}}E_{0} \end{array}$$

The total radial action can be rewritten as

(53) $$\begin{array}{c} {\tilde{R}}^{\pm }=\left(1+\sum_{i=1}^{\infty }\frac{\beta_{i}\hbar^{i}}{S_{bh}}\right)R_{0}^{\pm } \end{array}$$

The proportionality coefficients $\alpha_{i}$ and $\beta_{i}$ in Equations (52) and (53) are both dimensionless. To modify the entropy of this black hole, we can express the scaling factors of $R_{i}^{\pm }$ and $R_{0}^{\pm }$ as $\frac{\beta_{i}\hbar^{i}}{S_{bh}}$. Therefore, according to the first law of black hole thermodynamics, the equation satisfied by the Bekenstein–Hawking entropy ${\tilde{S}}_{BH}$ of this black hole with *ℏ* perturbation corrections is approximately

(54) $$\begin{array}{cc} d{\tilde{S}}_{BH} & =\frac{dM-\Omega dJ-QdV}{T_{H}}{\left(1+\sum_{i=1}^{\infty }\frac{\beta_{i}\hbar^{i}}{S_{bh}}\right)}^{-1}\\ \; & =\frac{dM-\Omega dJ-QdV}{T_{h}}{\left(\frac{1+2a^{\prime }+2\lambda c_{t}^{2}}{1+2a^{\prime }+2\lambda c_{r}^{2}}\right)}^{\frac{1}{2}}\left(1-\sum_{i=1}^{\infty }\frac{\beta_{i}\hbar^{i}}{S_{bh}}\right) \end{array}$$

where $T_{h}$ is the Hawking temperature at the event horizon of the black hole before Lorentz-breaking corrections are applied. Rewrite Equation (39) as

(55) $$\begin{array}{c} T_{H}=T_{h}\left(\frac{1+2a^{\prime }+2\lambda c_{r}^{2}}{1+2a^{\prime }+2\lambda c_{t}^{2}}\right) \end{array}$$

Integrate both sides of Equation (54), noting $T_{h}d\;S_{\mathrm{bh}}=dM-\Omega dJ-QdV$, and we obtain

(56) $$\begin{array}{cc} {\tilde{S}}_{BH} & =\int dS_{bh}{\left(\frac{1+2a^{\prime }+2\lambda c_{t}^{2}}{1+2a^{\prime }+2\lambda c_{r}^{2}}\right)}^{\frac{1}{2}}\left(1-\sum_{i=1}^{\infty }\frac{\beta_{i}\hbar^{i}}{S_{bh}}\right)\\ \; & ={\left(\frac{1+2a^{\prime }+2\lambda c_{t}^{2}}{1+2a^{\prime }+2\lambda c_{r}^{2}}\right)}^{\frac{1}{2}}\left(S_{bh}-\beta_{i}\hbar^{i}\ln S_{bh}+\cdots \right) \end{array}$$

The first term on the right-hand side of this equation represents the Bekenstein–Hawking entropy in the semiclassical theory of gravity, while the second term and subsequent terms represent the results of quantum perturbation theory corrections. It is evident that the black hole entropy after quantum corrections is not only dependent on the results of semiclassical theory but also on the Planck constant.

## 4. Discussion

Based on the research above, the Bekenstein–Hawking entropy of this black hole, denoted as $S_{bh}$ in Equation (47), represents the entropy without any modifications. When considering the corrections due to Lorentz-breaking fermionic Einstein–aether theory, the Bekenstein–Hawking entropy of this black hole, denoted as $S_{BH}$, is derived as shown in Equation (48). Furthermore, incorporating quantum perturbation correction theories leads to the Bekenstein–Hawking entropy of this black hole, denoted as ${\tilde{S}}_{BH}$, as given in Equation (56). The investigation of Bekenstein–Hawking entropy for different black holes remains a meaningful endeavor, emphasizing the ongoing relevance of research into topics such as black hole entropy, black hole information, and black hole thermodynamics. Equation (14) is a modified form of the dynamics equation for spin-1/2 fermions in semiclassical theory, which includes Lorentz-breaking correction terms. By studying this equation in the spacetime of the black hole, we obtain Hawking temperature $T_{H}$ and Bekenstein-Hawking entropy $S_{BH}$ of the HNUTKN black hole, which are results of semiclassical theory that include the effects of Lorentz-breaking corrections. Equations (53) and (56) both consider the results after quantum corrections, which go beyond semiclassical theory. Clearly, the entropy of this black hole is not only related to $a^{\prime }$, λ, $C_{t}$, and $C_{r}$, but also to $\hbar^{i}$. Equation (56) is the modified expression for the entropy of this black hole. The black hole studied in this paper possesses the characteristics of a general stationary black hole. Therefore, the series of results obtained above include relevant results for specific cases such as Kerr black holes and Kerr–Newman–de Sitter black holes. The HNUTKN black hole also has a cosmological horizon. If we want to study the quantum tunneling radiation characteristics at the cosmological horizon of the black hole, we only need to replace $r_{H}$ with $r_{C}$ in the research methods and the series of results obtained above. From the perspective of quantum tunneling radiation, the spatial region that the radiation reaches is $r>r_{H}$ at the event horizon of this black hole, while the spatial region that the radiation reaches is $r<r_{C}$ at the cosmological horizon of this black hole. Further discussion is needed on the physical significance of Equation (35). From the perspective of quantum tunneling radiation, $\omega_{0}$ represents the chemical potential, which is formed by the rotation and charge of the black hole. It includes the electromagnetic potential and the rotational potential, thus determining the quantum tunneling radiation characteristics of this black hole. From the perspective of quantum non-thermal radiation, $\omega_{0}$ is the maximum value of the particle Dirac energy level crossing, and this maximum value determines the condition for quantum non-thermal radiation to occur as $m<\omega ⩽\omega_{0}$. If we want to study the quantum non-thermal radiation of this black hole, the mathematical expression of the Dirac energy level must include contributions from the Chiral correction term $b^{\prime }$.

It should be further noted that the above research method and conclusions are specific to the HNUTKN black hole, and, for different types of black holes and fermions with different spins, specific analyses and research are required. For bosons, the above method cannot be used to study the modification of black hole entropy, and the modification of the scalar field action by Lorentz-breaking theory should be considered first. After modifying the boson dynamic equation on this basis, the particular significance of the black hole entropy can be analyzed.

## Data Availability

The data presented in this study are available on request from the corresponding author.
